# PLCE1 regulates the migration, proliferation, and differentiation of podocytes

**DOI:** 10.1038/s12276-020-0410-4

**Published:** 2020-04-01

**Authors:** Seyoung Yu, Won-Il Choi, Yo Jun Choi, Hye-Youn Kim, Friedhelm Hildebrandt, Heon Yung Gee

**Affiliations:** 1 Department of Pharmacology, Brain Korea 21 PLUS Project for Medical Sciences, Yonsei University College of Medicine, Seoul, 03722 Korea; 2 Department of Medicine, Division of Nephrology, Boston Children’s Hospital, Harvard Medical School, Boston, MA 02115 USA

**Keywords:** Mechanisms of disease, Focal segmental glomerulosclerosis

## Abstract

*PLCE1* encodes phospholipase C epsilon, and its mutations cause recessive nephrotic syndrome. However, the mechanisms by which PLCE1 mutations result in defects associated with glomerular function are not clear. To address this, we investigated the function of PLCE1 in podocytes called glomerular epithelial cells, where the pathogenesis of nephrotic syndrome converges. PLCE1 colocalized with Rho GTPases in glomeruli. Further, it interacted with Rho GTPases through the pleckstrin homology domain and Ras GTP-binding domains 1/2. Knockdown or knockout of *PLCE1* in podocytes resulted in decreased levels of GTP-bound Rac1 and Cdc42, but not those of RhoA, and caused a reduction in cell migration. PLCE1 interacted with NCK2 but not with NCK1. Similar to the *PLCE1* knockout, *NCK2* knockout resulted in decreased podocyte migration. Knockout of *PLCE1* reduced the EGF-induced activation of ERK and cell proliferation in podocytes, whereas knockout of *NCK2* did not affect proliferation. Further, the knockout of *PLCE1* also resulted in decreased expression of podocyte markers, including *NEPH1*, *NPHS1*, *WT1*, and *SYNPO*, upon differentiation, but the knockout of *NCK2* did not affect the expression of these markers. Therefore, our findings demonstrate that PLCE1 regulates Rho GTPase activity and cell migration through interacting with NCK2 and that PLCE1 also plays a role in the proliferation and differentiation of podocytes, regardless of the presence of NCK2.

## Kidney disease: The flaw behind a faulty filter

A genetic mutation associated with kidney disease impairs the maturation and migration of cells that filter waste products out of the blood. Tiny tendrils from kidney cells called podocytes establish a tight meshwork that keeps blood proteins in circulation while allowing unwanted contaminants to pass through. Mutations in the *PLCE1* gene disrupt this filter, leading to a disorder called nephrotic syndrome Researchers led by Heon Yung Gee at Yonsei University College of Medicine, Seoul, South Korea, have uncovered mechanisms underlying this malfunction. Working with cultured podocytes, they showed that loss of *PLCE1* impairs cell migration, potentially undermining their ability to form a meshwork. The researchers also found that the protein encoded by *PLCE1* interacts with other molecules that promote cell division and maturation, revealing another mechanism by which mutations could contribute to loss of podocyte function.

## Introduction

Filtration is an important function of the kidneys, and the human kidney contains one million glomeruli (filtering units). Glomeruli consist of three cell types: podocytes, endothelial cells, and mesangial cells^1^. Podocytes are neuron-like terminally differentiated epithelial cells that contain three segments: the cell body, major processes, and foot processes. The foot processes are in direct contact with the urinary side of the glomerular basement membrane and interdigitate with the foot processes from neighboring cells through specialized intercellular junctions (the ‘slit diaphragm’), thereby forming sieve-like structures that prevent the loss of plasma proteins into the urine^1^. This structure serves to create a maximal filtration space between cells while simultaneously supporting and maintaining the glomerular basement membrane^2^.

Defects in the glomerular filter result in nephrotic syndrome, a kidney disease characterized by albumin loss (proteinuria). Nephrotic syndrome is classified as either steroid-sensitive or steroid-resistant, depending on the response to steroids, which are commonly used as first-line therapy^3^. A significant proportion of steroid-resistant nephrotic syndromes (SRNSs) have a genetic background and result from a single-gene defect. The genetic heterogeneity of SRNS can be demonstrated by the fact that mutations in approximately 50 genes have been identified to be capable of causing SRNS^4,5^. Research on the function of genes linked to SRNS has revealed that podocytes are critical sites for the pathogenesis of SRNS^6,7^. SRNS is accompanied by the rearrangement of the actin cytoskeleton and simplification of interdigitating cellular processes of podocytes, called foot process effacement, which is a classic feature of proteinuric kidney diseases, including SRNS^7^.

Mutations in *PLCE1*, the gene encoding phospholipase C epsilon 1, have been described in patients with early-onset nephrotic syndrome^8^. In individuals exhibiting *PLCE1* mutations, kidney histology shows diffuse mesangial sclerosis (DMS) in most cases and focal segmental glomerulosclerosis (FSGS) in others^9^. Members of the phosphoinositide-specific phospholipase C family catalyze the hydrolysis of membrane phospholipids to generate the second messenger molecules inositol 1,4,5-trisphosphate (IP_3_) and diacylglycerol (DAG), which initiate intracellular cell growth and differentiation pathways^10^. PLCE1 is activated by G proteins, such as G_α12/13_, and small GTPases, such as Ras and RhoA^11,12^. DAG is an agonist of TRPC6, whose gain-of-function mutations cause late-onset nephrotic syndrome^13,14^. Therefore, it was shown that loss of PLCE1 can lead to disturbed DAG-mediated TRPC6 activation. However, podocytes isolated from *Plce1*^−/−^ mice were indistinguishable from control podocytes with respect to TRPC6 activation, indicating a redundancy in the PLC function in podocytes in this particular context^15^.

The role of PLCE1 in podocytes and the pathogenesis of nephrotic syndrome due to the loss of PLCE1 are not well understood. To address this, PLCE1-deficient podocytes were examined, and it was found that the migration, proliferation, and maturation of podocytes were negatively affected by the loss of PLCE1, thereby revealing a link between PLCE1 mutations and podocytopathy.

## Materials and methods

### Plasmids, cell culture, and transfection

The human PLCE1 clone has been described previously^8^. The fragments of PLCE1 were subcloned into pEGFP-C1 (Clontech, Mountain View, CA, USA). NCK1 and NCK2 clones were purchased from the Harvard PlasmID Database. Immortalized human podocytes were provided by Moin Saleem (University of Bristol, Bristol, UK) and maintained in RPMI plus GlutaMAX-I (Gibco) supplemented with 10% FBS, penicillin–streptomycin (50 IU/ml and 50 μg/ml, respectively), and insulin-transferrin-selenium-X. Plasmids and siRNA were transfected into podocytes cultured at a permissive temperature of 33 °C using Lipofectamine 2000 (Invitrogen, Carlsbad, CA, USA). Podocytes were cultured for differentiation at 37 °C for 14 days. The *PLCE1*-specific and control scrambled siRNAs were purchased from Qiagen. HEK293 or Lenti-X 293 T cells were propagated in DMEM supplemented with 10% FBS and penicillin–streptomycin (50 IU/ml and 50 μg/ml, respectively). sgRNAs targeting human *NCK2* (sgRNA1, 5’-GCTATGTACCGTCCAACTACG-3’; sgRNA2, 5’-GTGGACATCAAGAAGAACGAG-3’; and sgRNA3, 5’-GTACCGTCCAACTACGTGGAG-3’) and human *PLCE1* (sgRNA1, 5’-GCCAGCCTCCGAGACAGCCCA-3’; sgRNA2, 5’-GCCCATGGAAGGATAAGCGT-3’; and sgRNA3, 5’-GACTTCTTGCTCCCAAAGACG-3’) were cloned into BsmBI-digested lentiCRISPR v2 (Addgene #52961, Watertown, MA, USA). To establish *PLCE1* or *NCK2* knockout cell lines, lentiCRISPR v2, pMD2.G, and psPAX2 were transfected into Lenti-X 293T cells (Clontech). The supernatant containing lentivirus was collected 48 h after transfection and passed through a 0.2 µm filter. Cultured podocytes were transduced with lentivirus containing lentiCRISPR clones, selected, and maintained in the presence of 4 μg/ml puromycin.

### Immunoblotting, GST pulldown, and immunoprecipitation

These experiments were performed as described previously^16^. GST-Rac1 (Addgene, #12200) and GST-RhoA (Addgene, #12202) were purified from the BL21 (DE3) *E. coli* strain, and GST-PAK1 CRIB and GST-rhotekin RBD domains were purchased from Cytoskeleton, Inc. Anti-FLAG, anti-Myc, anti-p-ERK, anti-p-JNK, anti-ERK (Cell Signaling Technology, Danvers, MA, USA), anti-Rac1, anti-Cdc42 (BD Transduction Laboratories), anti-RhoA (Santa Cruz Biotechnology, Dallas, TX, USA), and anti-GFP (Invitrogen) were purchased from the indicated commercial sources. The anti-PLCE1 antibody has been previously described by Hinkes et al.^8^. Coimmunoprecipitation was performed using EZview Red anti-FLAG M2 or Anti-c-Myc Affinity Gels (Sigma-Aldrich, St.Louis, MO, USA). The intensities of immunoblots were analyzed using ImageJ.

### Immunofluorescence

The rat kidney tissue was fixed in 4% paraformaldehyde overnight at 4 °C, embedded in paraffin blocks, and cut into 5 μm-thick sections. The sections were then mounted on slides, deparaffinized, and rehydrated through a graded series of ethanol concentrations. After rehydration, antigen retrieval was performed by boiling the sections for 30 min using a Retrieve-All Antigen unmasking system 1 (pH 8; BioLegend, San Diego, CA, USA). Sections were permeabilized with 1% sodium dodecyl sulfate for 10 min at room temperature, blocked with normal donkey serum (1% BSA, 10% donkey serum in PBS) for 1 h, and incubated overnight with primary antibodies. The next day, sections were washed with PBS and incubated with secondary antibodies, Alexa 488 and Alexa 594 (Invitrogen); the sections were then washed with PBS, stained with DAPI and mounted on coverslips. Fluorescence images were obtained using an LSM 780 confocal microscope (Carl Zeiss).

### Wound-healing assay

Cells were seeded in 96-well image lock plates (Essen Bioscience, Ann Arbor, MI, USA). Podocytes were examined for confluency as a monolayer via light microscopy before the initiation of the wound-healing assay. Scratches were made using a 96-pin tool (Woundmaker), according to the protocol provided by the manufacturer.

### Podocyte migration and proliferation assays

Real-time migration or proliferation assays were performed using the xCELLigence system (ACEA Biosciences, Inc, San Diego, CA, USA), according to the manufacturer’s instructions. Briefly, 2 × 10^4^ cells were seeded in an E-Plate for the proliferation assay and were treated with 100 ng/ml epidermal growth factor. For the migration assay, 4 × 10^4^ cells were seeded in serum-free media in the upper chamber of a CIM-Plate 16, while the lower chambers were filled with 10% FBS for chemoattraction or with serum-free media. The data were analyzed using RTCA software. The results are presented as the time-versus-cell index.

### Quantitative RT-PCR

Total RNA was extracted from podocytes and reverse-transcribed using the iScriptTM Select cDNA Synthesis Kit (Bio-Rad, Hercules, CA), according to the manufacturer’s instructions. Target amplification was performed in 96-well plates using the StepOnePlus^TM^ Real-Time PCR System and SYBR Green PCR Master Mix (Thermo Fisher Scientific, San Jose, CA, USA), according to the manufacturer’s instructions. The primers used in the RT-PCR analysis were *GAPDH* (5’-CATCAGCAATGCCTCCTG-3’, 5’-AGTCCTTCCACGATACCAAAG-3’), *NEPH1* (5’-GCCATCTACTCGTCGTTTAAG-3’, 5’-GCACGGTAGTCAGCATAGAG-3’), *NPHS1* (5’-CCTTCTGCAAGTGTCATCC-3’, 5’-GTTAGCGGACACGGACAC-3’), *SYNPO* (5’-AAGTCACATCCAGCTCCTTC-3’, 5’-CTTCTCCGTGAGGCTAGTG-3’), and *WT1* (5’-CCCTACAGCAGTGACAATTTATAC-3’, 5’-TGCCCTTCTGTCCATTTC-3’). All samples were run in triplicate. The relative RNA expression levels were calculated via a comparative threshold cycle (Ct) method using GAPDH as the control: ΔCt = Ct(GAPDH)-Ct(target gene). The fold change in the gene expression, which was normalized to GAPDH and relative to the control sample, was calculated as 2^−ΔΔCt^.

### Statistical analysis

The results are presented as the means ± SEM or SD for the indicated number of experiments. Statistical analysis of continuous data was performed with a 2-tailed Student’s *t*-test or a Mann–Whitney *U* test, as appropriate. *P* < 0.05 was considered statistically significant.

## Results

### PLCE1 interacts with RhoA and Rac1 in podocytes

**Fig. 1:**
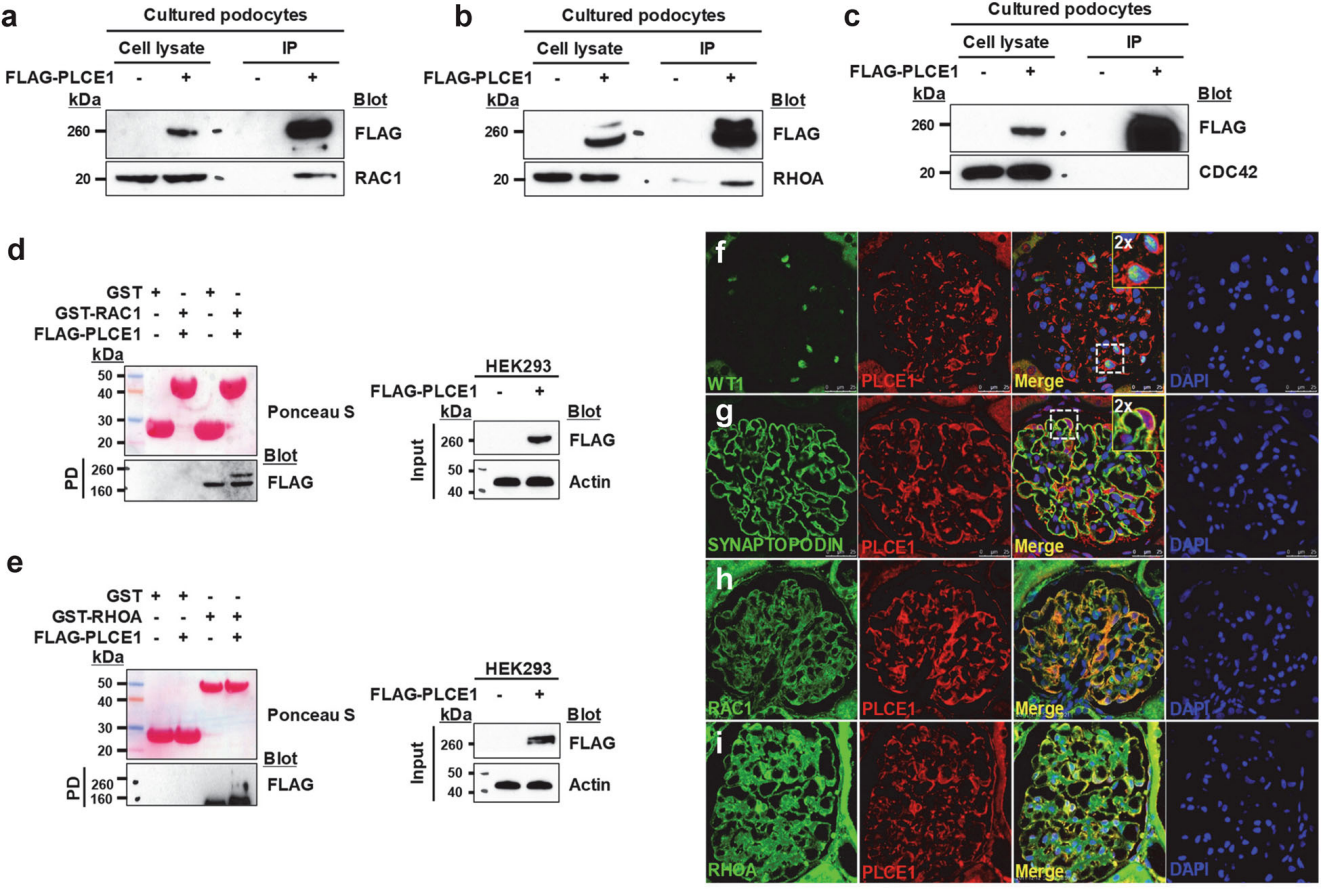
PLCE1 interacts with Rac1 and RhoA. **a**–**c** Immunoprecipitation of PLCE1 with Rho GTPase, Rac1 (**a**), RhoA (**b**), and Cdc42 (**c**) in cultured podocytes. Immunoblotting showed that PLCE1 interacts with Rac1 and RhoA but not with Cdc42. **d**–**e** FLAG-PLCE1 expressed in HEK293 cells was precipitated by GST- Rac1 (**d**) and GST-RhoA (**e**). Ponceau red staining at the top shows the GST proteins used. **f**–**i** Costaining of PLCE1 with WT1 (**f**), SYNAPTOPODIN (**g**), Rac1 (**h**) and RhoA (**i**) using immunofluorescence in adult rat glomeruli. PLCE1 colocalized with Rac1 and RhoA in podocyte cell bodies. Immunoprecipitation (IP) and pulldown (PD) experiments are representative of more than three experiments.

PLCE1 is activated by the direct binding of small GTPases, including Ras and RhoA^11,12^. As Rac1, Cdc42, and RhoA are implicated in the pathogenesis of nephrotic syndrome in mice and humans^16–20^, we examined whether these Rho GTPases interact with PLCE1 in cultured podocytes. Coimmunoprecipitation in the background of PLCE1 overexpression revealed that PLCE1 interacts with Rac1 (Fig. 1a) and RhoA (Fig. 1b) but not with Cdc42 (Fig. 1c). The interaction of PLCE1 with Rac1 and RhoA was further confirmed by the pulldown assay (Fig. 1d, e).

The expression of PLCE1 in the glomeruli was examined. PLCE1 has been reported to be expressed in developing and mature podocytes^8^, and we also confirmed that PLCE1 is enriched in podocyte cell bodies and processes by staining for the podocytic markers WT1 and SYNAPTOPODIN using immunofluorescence (Fig. 1f, g). Immunostaining of PLCE1 with Rac1 showed the colocalization of both proteins in cell bodies and the processes of podocytes (Fig. 1h), whereas PLCE1 colocalized with RhoA mostly in the cell bodies (Fig. 1i).

**Fig. 2:**
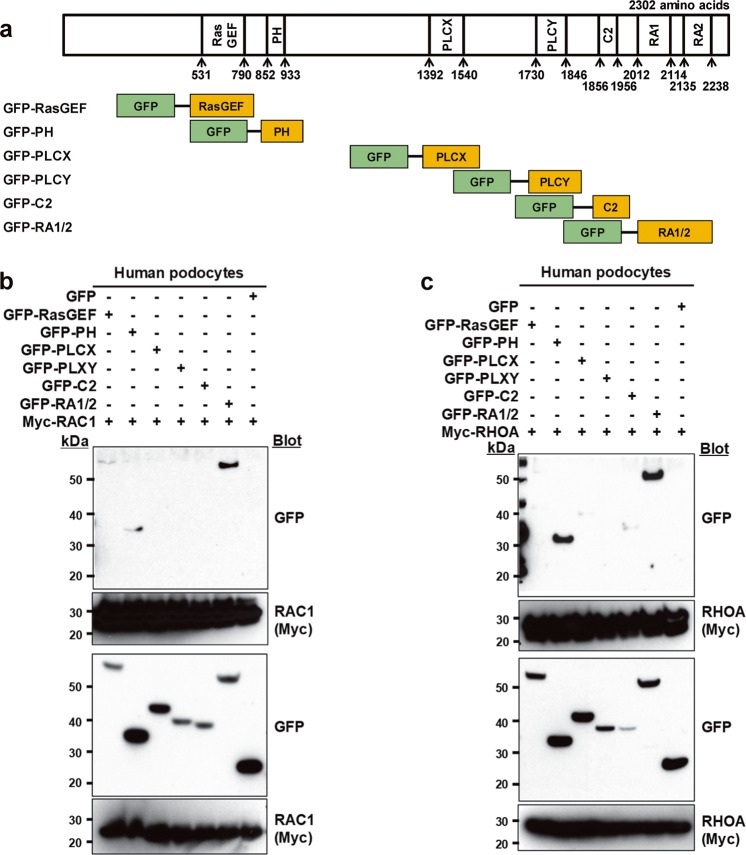
The PH and RA1/2 domains of PLCE1 are required for the interaction with Rac1 and RhoA. **a** Schematic representation of the PLCE1 domains. PLCE1 contains a RasGEF domain, a pleckstrin homology (PH) domain, two regions of sequence among PLCs (X and Y), a C2 domain, and two Ras-binding (RA) domains. The numbers represent the amino acid positions. The fragments of PLCE1 were fused to GFP at their N-terminus. **b**, **c** Interactions of PLCE1 domains with Rho GTPase Rac1 and RhoA. GFP-tagged PLCE1 fragments and Myc-tagged Rac1 (**b**) and RhoA (**c**) were transfected into podocytes, and immunoprecipitation was performed using anti-Myc agarose beads. Notably, both RAC1 and RhoA bind to PLCE1 through the PH and RA1/2 domains.

To identify the domain of PLCE1 that interacts with Rac1 and RhoA, coimmunoprecipitation was performed in cultured podocytes (Fig. 2). PLCE1 contains a RasGEF (a.k.a., CDC25, guanine nucleotide exchange factor for Ras-like small GTPases) domain, a pleckstrin homology (PH) domain, two regions with high sequence conservation among all PLCs (X and Y), a C2 domain, and two Ras-binding (RA) domains (Fig. 2a)^12^. Among these, both Rac1 and RhoA bind to PLCE1 through the PH and RA1/2 domains (Fig. 2b, c).

### Loss of PLCE1 resulted in decreased active Rac1 and Cdc42 and decreased podocyte migration

**Fig. 3:**
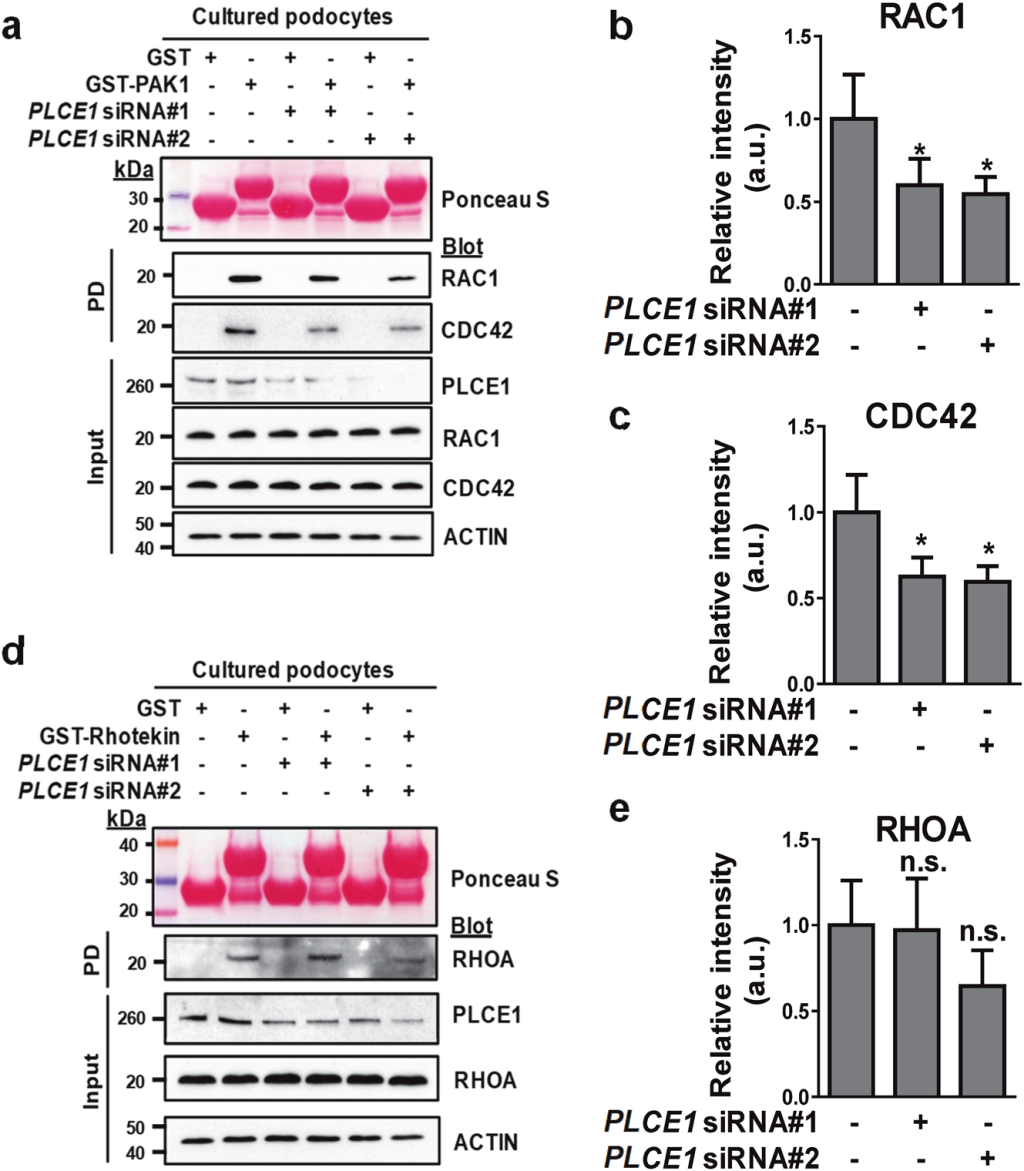
The effects of the PLCE1 knockdown on Rho GTPase activity. **a** Active GTP-bound Rac1 and Cdc42 precipitated from podocytes transfected with *PLCE1* siRNA using a GST-PAK1 (CRIB domain) pulldown assay. Ponceau red staining at the top shows the GST proteins used. Compared with control podocytes, podocytes transfected with *PLCE1* siRNA exhibited a significant decrease in GTP-bound Rac1 and Cdc42 levels. **b**, **c** Quantification of Rac1 (**b**) and Cdc42 (**c**) in *PLCE1* knockdown cells compared to that in control cells. **d** Active GTP-bound RhoA precipitated from podocytes transfected with *PLCE1* siRNA using a GST-rhotekin (RBD domain) pulldown assay. Cells transfected with or without *PLCE1* siRNA exhibited no significant differences in GTP-bound RhoA. **e** Quantification of RhoA in *PLCE1* knockdown cells compared with that in control cells. Error bars indicate the standard deviations for more than three independent experiments. *P < 0.05; n.s. not significant.

As PLCE1 interacts with Rac1 and RhoA in podocytes, the effect of PLCE1 on the active states of Rho GTPases was examined. *PLCE1* was knocked down using siRNAs, and pulldown assays were performed using purified PAK1 and Rhotekin. Knockdown of *PLCE1* in cultured podocytes led to a decrease in active GTP-bound Rac1 (43%) and Cdc42 (40%) (Fig. 3a–c) but had no effect on the levels of active RhoA in the cultured podocytes (Fig. 3d, e).

**Fig. 4:**
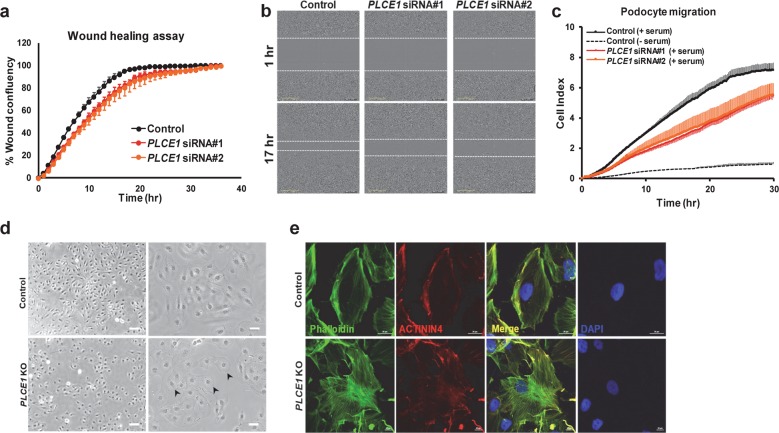
Depletion of PLCE1 resulted in decreased podocyte migration and changed cell morphology. **a** Wound closure was reduced in podocytes transfected with *PLCE1* siRNAs (red and orange dots) compared to that in control podocytes (black dots). **b** Movement of cells into the wound is shown at 1 and 17 h after scratching. *PLCE1* knockdown podocytes showed delayed wound closure. **c** The effect of *PLCE1* knockdown on serum-induced podocyte migration. Podocytes transfected with *PLCE1* siRNA (red and orange lines) exhibited decreased migration compared to that of the control podocytes (solid black line). Error bars are shown in only one direction for clarity and indicate standard deviations for more than three independent experiments. **d**, **e** The effect of *PLCE1* knockout (KO) on podocyte morphology. *PLCE1* KO resulted in the loss of terminal arborizations in undifferentiated podocytes (**d**) and an increased number of stress fibers in differentiated *PLCE1* KO podocytes relative to that in control podocytes (**e**).

As Rho GTPases regulate cell migration^21^ and *PLCE1* knockdown alters the active status of Rac1 and Cdc42, podocyte migration was examined using two different approaches. In the wound-healing assay, podocytes with siRNA-mediated *PLCE1* knockdown exhibited delayed wound closure compared to the control podocytes (Fig. 4a, b). This result is consistent with the findings of Rao et al.^5^. In addition, the Boyden chamber assay using the xCELLigence system showed that the knockdown of *PLCE1* resulted in a decreased migratory phenotype in cultured podocytes (Fig. 4c).

As Rho GTPases play an important role in organizing the actin cytoskeleton, thereby affecting the cell morphology^21^, we examined podocyte morphology in the background of the *PLCE1* knockout (KO) (Supplementary Fig. 1). *PLCE1* KO resulted in the loss of terminal arborizations in undifferentiated podocytes (Fig. 4d). Furthermore, differentiated *PLCE1* KO podocytes displayed increased stress fibers, reflecting damage to the podocyte architecture (Fig. 4e)^22^.

Taken together, the loss of PLCE1 resulted in decreased active Rac1 and Cdc42, thereby leading to a reduced migratory phenotype in cultured podocytes.

### PLCE1 interacts with NCK2 in podocytes

**Fig. 5:**
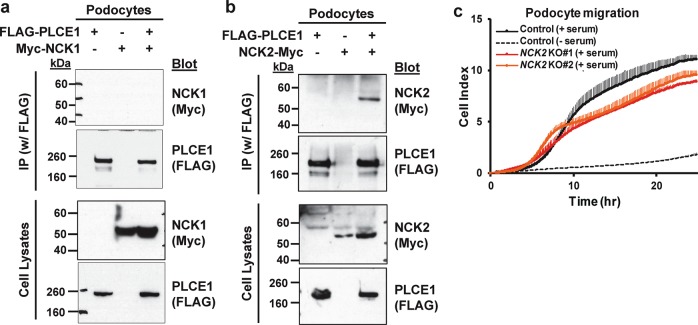
NCK2 interacts with PLCE1 and affects the migration in podocytes. **a**, **b** Interaction of PLCE1 and NCKs. The plasmids containing FLAG-tagged PLCE1 and Myc-tagged NCKs were transfected into podocytes, and cell lysates were immunoprecipitated using anti-FLAG agarose beads. Notably, PLCE1 interacted with NCK2 (**b**) but not with NCK1 (**a**). The immunoblots are representative of more than three experiments. IP, immunoprecipitation. **c** The effect of *NCK2* knockout (KO) on podocyte migration. NCK2-depleted podocytes exhibited decreased migration (red and orange lines) compared with that of the control podocytes (solid black line). Error bars are shown in only one direction for clarity and indicate standard deviations for more than three independent experiments.

NCK proteins, NCK1 and NCK2, are ubiquitously expressed adaptors that contain three Src homology 3 (SH3) domains and an SH2 domain; a podocyte-specific deletion of both genes leads to FSGS^23^. Coimmunoprecipitation was performed to investigate the interaction between PLCE1 and NCKs; this was based on the prediction of a web-based protein-protein interaction database, PrePPI (https://bhapp.c2b2.columbia.edu/PrePPI/), which showed that PLCE1 can interact with NCKs. The results showed that PLCE1 interacted with NCK2 but not with NCK1 (Fig. 5a, b).

NCKs relay signals from receptors at the plasma membrane to the actin cytoskeleton by simulating N-WASP-Arp2/3-induced actin nucleation^24^ or by activating Pak1^25^. As NCK2 interacts with PLCE1, the effect of NCK2 on podocyte migration was examined. Therefore, NCK2 was knocked out in cultured podocytes using the CRISPR/Cas9 system (Supplementary Fig. 2). *NCK2* knockout (KO) podocytes exhibited decreased migration in a manner similar to podocytes with *PLCE1* knockdown (Fig. 5c). These results suggest that PLCE1 cooperates with NCK2 to regulate the actin cytoskeleton in podocytes.

### PLCE1 regulates the activity of mitogen-activated protein kinases in podocytes

**Fig. 6:**
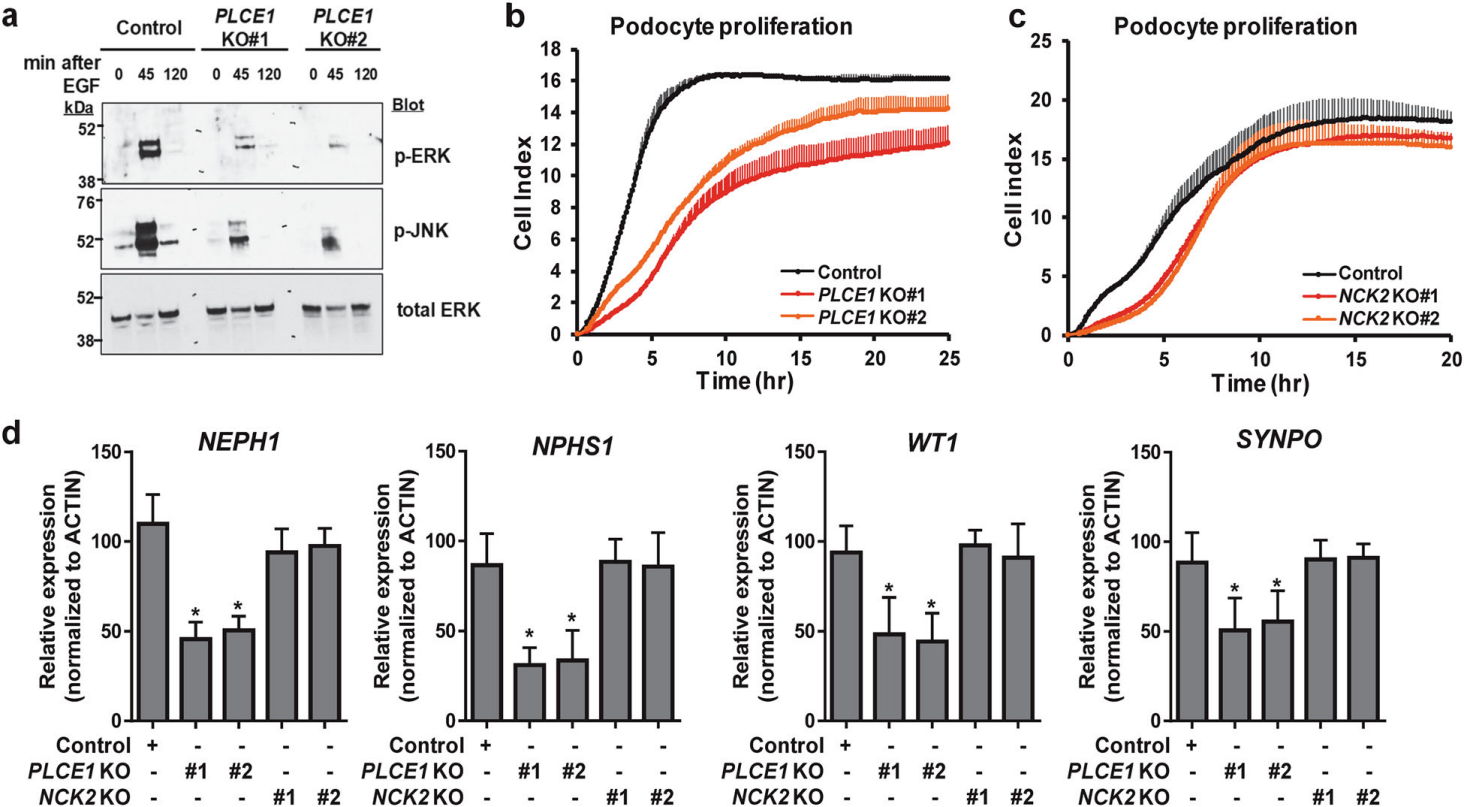
PLCE1 regulates mitogen-activated protein kinases (MAPKs) in podocytes. **a** MAPK phosphorylation was induced by 20 ng/µl epidermal growth factor (EGF) treatment for 45 or 120 min. EGF-induced phosphorylation of ERK and JNK was significantly reduced in *PLCE1* knockout (KO) podocytes. **b**, **c** The effects of PLCE1 or *NCK2* KO on podocyte proliferation. *PLCE1* KO podocytes exhibited lower proliferation (**b**, red and orange lines), whereas *NCK2* KO podocytes exhibited no significant differences in proliferation (**c**, red and orange lines) with respect to the control cells (black line). Error bars are shown in one direction only for clarity and indicate standard deviations from more than three independent experiments. **d** Expression analysis of podocyte markers. The expression of *NEPH1*, *NPHS1*, *WT1*, and *SYNPO* was measured by quantitative real-time PCR. The expression of podocyte markers was significantly reduced in *PLCE1* KO podocytes, whereas no difference in the levels of podocyte markers was observed between *NCK2* KO and control podocytes. Gene expression was normalized relative to that of actin. Data are presented as the mean ± standard deviation (*n* = 3). *P < 0.05.

PLCE1 transduces mitogenic signals through its activity as a RasGEF to stimulate the Ras and mitogen-activated protein kinase (MAPK) signaling pathway^11,26^. To investigate whether PLCE1 plays a similar function in podocytes, we investigated the Ras/MAPK signaling pathway. Upon stimulation of the control podocytes with epidermal growth factor (EGF), the levels of phosphorylated extracellular-signal-regulated kinase (ERK) and c-Jun N-terminal kinase (JNK) were increased after 45 min, but they returned to basal levels after 120 min (Fig. 6a). However, the EGF-induced phosphorylation of ERK and JNK was significantly reduced in *PLCE1* KO podocytes (Fig. 6a). In addition, *PLCE1* KO podocytes exhibited lower proliferation than the control podocytes (Fig. 6b). However, *NCK2* KO podocytes showed proliferation similar to that of control podocytes (Fig. 6c), indicating that NCK2 is not involved in the proliferation of podocytes.

The effect of *PLCE1* KO on podocyte differentiation was examined as MAPKs regulate differentiation^27^. When cultured at 37 °C for 14 days, the levels of podocyte markers, such as *NEPH1*, *NPHS1*, *WT1*, and *SYNPO*, were significantly reduced in the *PLCE1* KO podocytes compared to those in control podocytes, whereas no difference was observed between the *NCK2* KO and control podocytes with respect to differentiation (Fig. 6d).

Taken together, PLCE1 is involved in mediating the proliferation and differentiation of podocytes by regulating MAPKs independently of NCK2.

## Discussion

**Fig. 7:**
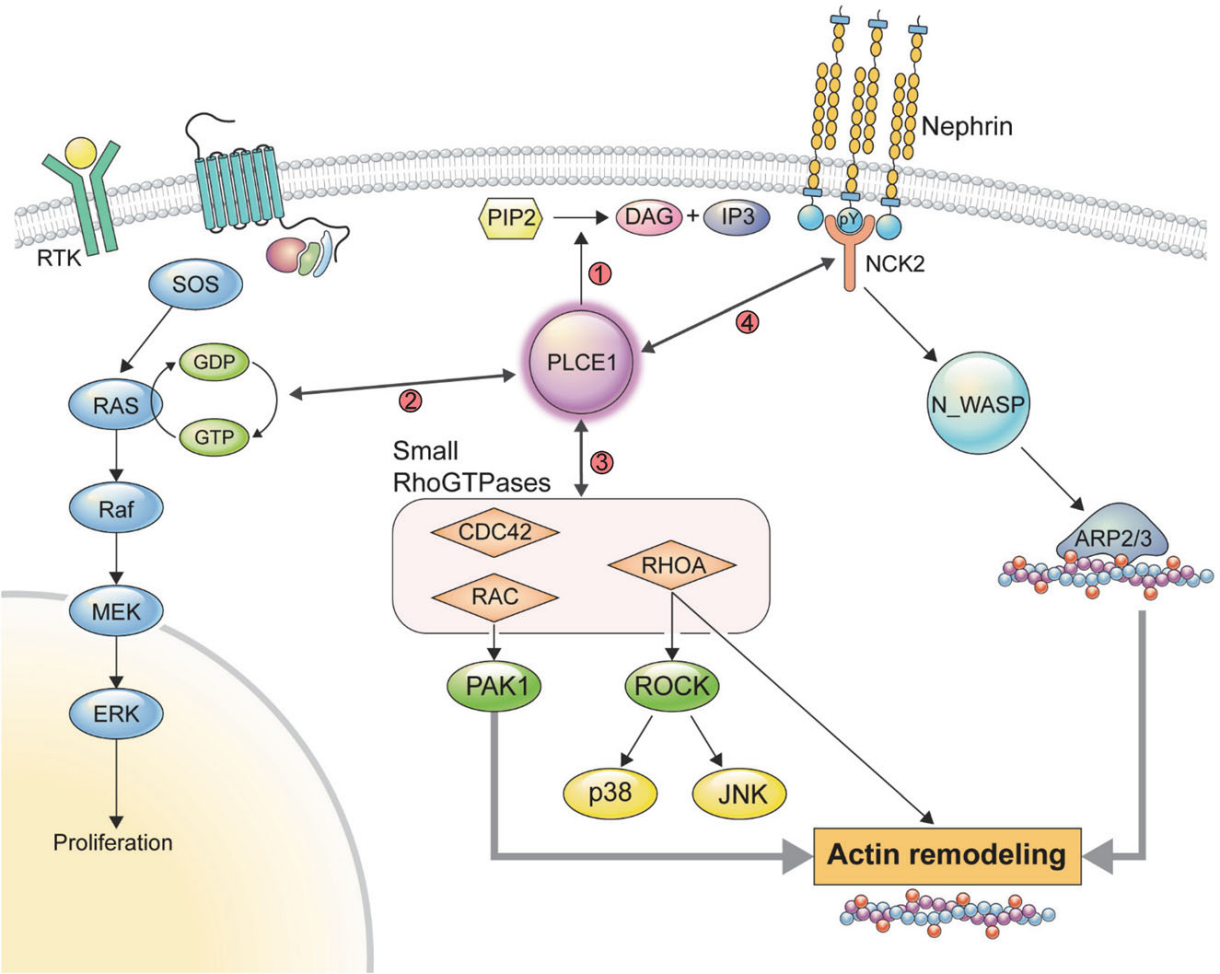
Functions of PLCE1 in podocytes. PLCE1 plays multiple roles in podocytes: (1) PLCE1 acts as a phospholipase and degrades phosphatidylinositol 4,5-bisphosphate (PIP_2_) into diacylglycerol (DAG) and inositol triphosphate (IP_3_). (2) PLCE1 can be activated by a G-protein and transduces mitogenic signals through its RasGEF activity to stimulate the Ras and MAPK pathways. (3) PLCE1 regulates the activity of Rho GTPases, which are the key regulators of actin dynamics and cell migration. Upon loss of PLCE1, the levels of active Rac1 and Cdc42 were reduced. (4) PLCE1 interacts with NCK2, which plays a pivotal role in stimulating N-WASP-Arp2/3-induced actin nucleation.

In this study, it was found that PLCE1 is involved in the regulation of Rho GTPases and MAPKs in podocytes (Fig. 7). It was also found that PLCE1 interacts with NCK2 but not with NCK1. *PLCE1* KO podocytes exhibit a decrease in active GTP-bound Rac1 and Cdc42 and decreased migration. In addition, *PLCE1* KO podocytes show a decrease in ERK phosphorylation and proliferation upon EGF stimulation and a decrease in the expression of podocyte markers upon differentiation.

PLCE1 is a multifunctional enzyme exhibiting phospholipase as well as RasGEF activities^26^. In podocytes, PLCE1 can be activated by the G_α_ protein upon the stimulation of the angiotensin II receptor by angiotensin II. Then, through its phospholipase activity, PLCE1 hydrolyzes phosphatidylinositol 4,5-bisphosphate, thereby generating DAG and IP_3_. DAG activates TRPC6, and IP3 activates a calcium channel located in the endoplasmic reticulum, thereby contributing to the increase in the intracellular Ca^2+^ concentration. In podocytes, PLCβ and PLCE1 play a redundant role in angiotensin II-induced DAG production, and angiotensin II-induced TRPC6 currents are normally generated in *Plce1*^−/−^ podocytes^15^. Indeed, PLCβ isoforms are essential for angiotensin II-mediated activation of TRPC6 in podocytes^28^. Taken together, the loss of phospholipase activity is not sufficient to explain the pathogenesis of nephrotic syndrome resulting from *PLCE1* mutations.

Rho GTPases are key players in the regulation of actin dynamics and cell migration^21^. Both increased and decreased Rho GTPase signaling interfere with podocyte mobility, thus causing proteinuria in humans and animal models^29^. RhoA directly activates the phospholipase activity of PLCE1^12^, but the active state of RhoA was not affected in *PLCE1* knockdown podocytes. However, it was found that the levels of GTP-bound Rac1 and Cdc42 were decreased by the *PLCE1* knockdown, which also led to a reduction in the migratory phenotype of podocytes. The podocyte-specific *Cdc42* KO mouse model exhibits severe proteinuria and early-onset FSGS, whereas the podocyte-specific *Rac1* KO mice are normal but are more susceptible to chronic hypertensive damage^17^. Therefore, decreased levels of active Cdc42 and Rac1 may have roles in *PLCE1* mutation-induced disease pathogenesis.

*PLCE1* mutations are histologically associated with DMS^8,9^, which is known to be characterized by a diffuse increased mesangial matrix and immature glomeruli^30^. In addition, podocytes are hypertrophic and have a cuboidal (immature) appearance^30^. In this study, *PLCE1* KO podocytes exhibited reduced MAPK activation and cell proliferation upon EGF stimulation. In addition, *PLCE1* KO podocytes expressed low levels of podocyte markers upon differentiation, indicating that they remain immature. These characteristics of *PLCE1* KO podocytes could explain the DMS observed upon analyzing the renal histology in individuals with *PLCE1* mutations.

NCK1 and NCK2 exhibited redundancy in podocytes, as the simultaneous deletion of both genes, but not that of a single gene, resulted in podocytopathy^23^. However, they also have distinct functions. For example, NCK1, but not NCK2, is ubiquitinated by c-Cbl, which competes with synaptopodin for binding to NCK1 and protects NCK1 from proteasomal degradation. Then, NCK1 induces the formation of stress fibers in cooperation with RhoA^31^. In this study, it was found that PLCE1 interacts with NCK2 but not with NCK1. *NCK2* KO resulted in the reduced migration of podocytes in a manner similar to that observed upon *PLCE1* knockdown. As the levels of active Rac1 and Cdc42 were reduced upon *PLCE1* knockdown, it will be interesting to examine whether NCK2 regulates Rac1 and Cdc42 along with PLCE1 in podocytes.

We revealed that PLCE1 plays multiple roles in podocytes. Mutational analysis of *PLCE1* revealed that mutations are distributed uniformly throughout *PLCE1*^32^. Some frameshift mutations at the C-terminus of *PLCE1* affect one or both of the RA domains, but the protein retains intact phospholipase and RasGEF activities. It was found that the RA domain is necessary for interacting with Rac1 and RhoA, suggesting that these mutations may be pathogenic as they dysregulate Rho GTPase signaling.

## Supplementary information

Supplementary Fig. 1-2
