# Supplementary material for: PLCE1 regulates the migration, proliferation, and differentiation of podocytes
Source: Exp Mol Med. 2020 Apr 1;52(4):594–603. doi: 10.1038/s12276-020-0410-4 (PMC7210307; doi:10.1038/s12276-020-0410-4)
Supplement: Supplementary file 1 — Supplementary Fig. 1-2 [file 12276_2020_410_MOESM1_ESM.pdf]

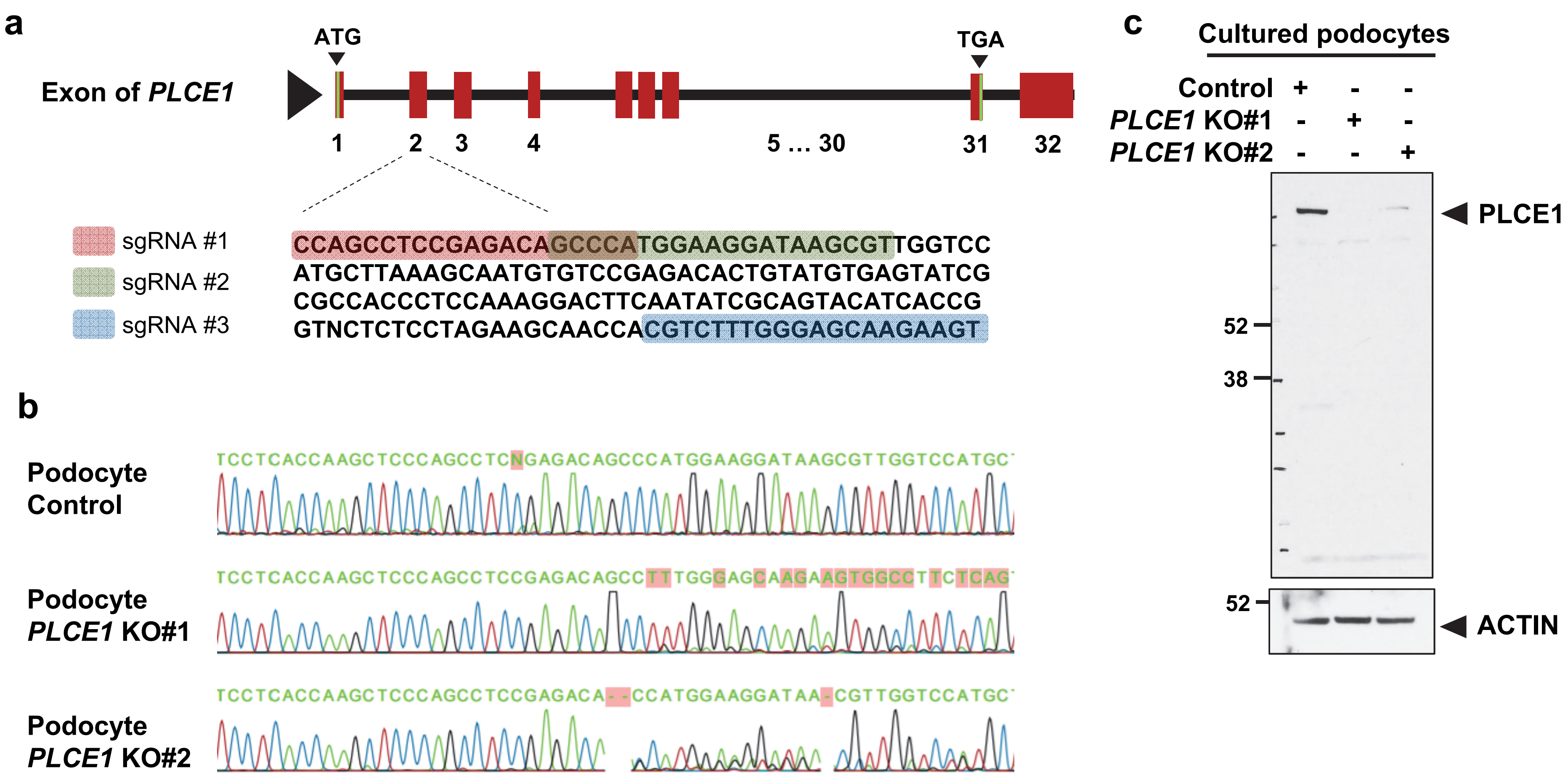

Supplementary Fig. 1 Generation of *PLCE1* knockout (KO) podocytes. a Three sgRNAs targeting the exon 2 of *PLCE1*. The each target DNA regions are indicated below pink-colored, green-colored, and blue-colored sequences, respectively. **b** Sanger sequencing of the exon 2 of *PLCE1* confirmed genome editing in podocytes. **c** Western blots of PLCE1 expression in the CRISPR/Cas9-edited KO cell lines.

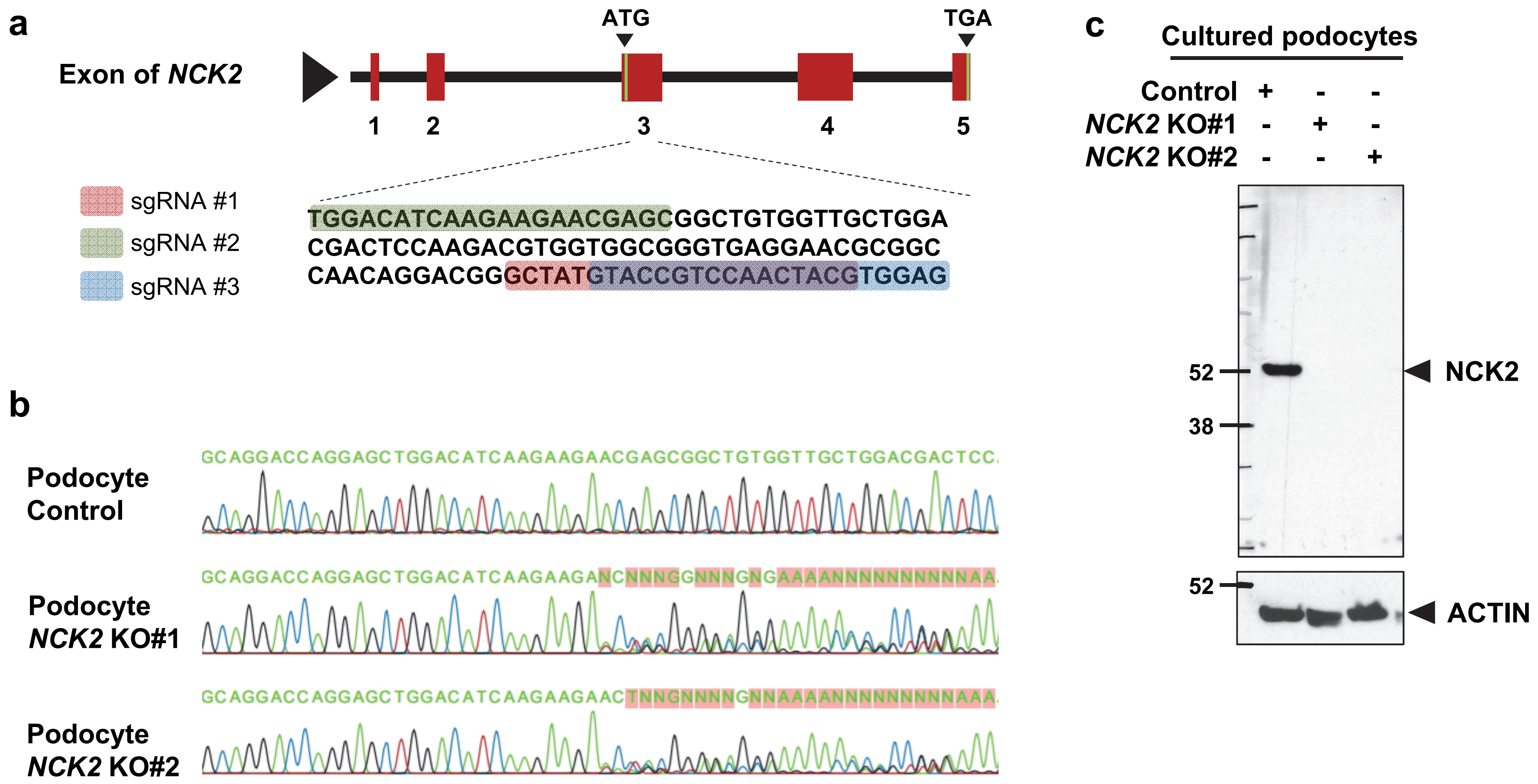

Supplementary Fig. 2 Generation of *NCK2* knockout (KO) podocytes. a Three sgRNAs targeting the exon 3 of *NCK2*. The each target DNA regions are indicated below pink-colored, green-colored, and blue-colored sequences, respectively. **b** Sanger sequencing of the exon 3 of *NCK2* confirmed genome editing in podocytes. **c** Western blots of NCK2 expression in the CRISPR/Cas9-edited KO cell lines.
